# Investment into Defensive Traits by Anuran Prey (*Lithobates pipiens*) Is Mediated by the Starvation-Predation Risk Trade-Off

**DOI:** 10.1371/journal.pone.0082344

**Published:** 2013-12-09

**Authors:** Amanda M. Bennett, David Pereira, Dennis L. Murray

**Affiliations:** Environmental and Life Sciences, Trent University, Peterborough, Ontario, Canada; Clemson University, United States of America

## Abstract

Prey can invest in a variety of defensive traits when balancing risk of predation against that of starvation. What remains unknown is the relative costs of different defensive traits and how prey reconcile investment into these traits when energetically limited. We tested the simple allocation model of prey defense, which predicts an additive effect of increasing predation risk and resource availability, resulting in the full deployment of defensive traits under conditions of high risk and resource saturation. We collected morphometric, developmental, and behavioural data in an experiment using dragonfly larvae (predator) and Northern leopard frog tadpoles (prey) subject to variable levels of food availability and predation risk. Larvae exposed to food restriction showed limited response to predation risk; larvae at food saturation altered behaviour, development, and growth in response to predation risk. Responses to risk varied through time, suggesting ontogeny may affect the deployment of particular defensive traits. The observed negative correlation between body size and activity level for food-restricted prey – and the absence of a similar response among adequately-fed prey – suggests that a trade-off exists between behavioural and growth responses when energy budgets are limited. Our research is the first to demonstrate how investment into these defensive traits is mediated along gradients of both predation risk and resource availability over time. The interactions we demonstrate between resource availability and risk level on deployment of inducible defenses provide evidence that both internal condition and extrinsic risk factors play a critical role in the production of inducible defenses over time.

## Introduction

There is an extensive conceptual framework for predicting how animals balance foraging activity and nutritional status against vulnerability to predators and exposure to predation risk [1], [2], [3]. The starvation–predation risk trade-off predicts that when food resources are limited, prey should act in a predation risk-prone manner and acquire requisite energy through foraging, but the cost of such a response is an increased exposure to predation risk [1]. In contrast, when food is abundant, prey should employ a predation risk-averse strategy by decreasing the probability of predator encounters at the cost of reduced foraging time. State-dependent models of investment in defense consider the starvation-predation risk trade-off from the perspective of an individual's condition (e.g. nutritional status) rather than through extrinsic risk factors [4]. Individual animals with a lower nutritional status should spend more time engaging in risky behaviour (e.g., foraging) compared to those with adequate energetic reserves. The simple allocation model predicts that animals with greater resource availability should invest surplus energy into predator avoidance or defense [5], [6], [7].

Sublethal effects of predation are not limited to behavioural responses and predation risk can also induce changes in prey morphology and life history. Plastic responses to predation risk can ultimately reduce fitness and alter population density; such effects are in addition to the direct effects of predation [8], [9], [10]. Alternate phenotypes that are designed to reduce predation risk may be more energetically costly to produce and maintain [8], [11], and the induction of defensive morphologies can result in reduced growth as well as lower lifetime reproductive output [12], [13]. Behavioural responses also are subject to the growth-predation risk trade-off, with reduced foraging activity leading to higher survivorship but at the cost of lower resource acquisition [14], [15], [16]. Therefore, when resources are limited, animals may not be able to mount the full suite of responses to predation risk, as behavioural plasticity must be considered in conjunction with plasticity in morphology and life history.

Selection should favour prey that appropriately balance behavioural and morphological plasticity in the face of predation risk. Increased vulnerability via the loss of a morphological defense (such as that occuring after defensive autotomy) alters behavioural anti-predator strategies, causing increased refuge use and reduced foraging success [17], [18], [19]. What remains unclear is whether the opposite holds true – whether prey that exhibit predator-induced morphological defenses also show riskier behaviour to increase foraging success. Energy limitation may restrict prey ability to modify foraging activity according to predation risk [2], [20]. Morphological variation may therefore be an important alternative response to reduce risk during predator encounters when behavioural responses are too costly, and it is likely that morphological plasticity is especially prevalent when food is restricted and behavioural responses are absent. Indeed, some models of larval defense investment predict that morphological responses should be insensitive to resource level, whereas behavioural responses will vary with resource availability relative to the cost of reduced growth rates and size at metamorphosis [21]. Alternatively, investment in morphological defense may be as energetically costly as a behavioural defense and morphological responses could therefore reflect resource availability to a similar extent. Predator-induced morphologies have been associated with higher energetic and developmental costs, such as reduced growth in body tissue [22] or shorter gut length and reduced digestion efficiency [23]. It follows that severe food-restriction should lead to neither behavioural nor morphological responses to predation risk, whereas prey with unrestricted access to resources should channel any excess energy into avoiding predation risk. Accordingly, we expect plastic responses in both behaviour and morphology among animals with abundant food resources.

Evaluation of investment into morphological versus behavioural defenses across a resource gradient in larval prey is further complicated by ontogeny. Growth and development rates can reflect responses to predation risk, optimal size at metamorphosis, optimal timing of metamorphosis, or any combination thereof [21]. Models with fixed size constraints at metamorphosis have predicted investment into both morphological and behavioural defenses should peak at intermediate resource levels [7]. However, prey may employ a strategy of maturing at a smaller body size rather than investing in morphological defenses if the benefit of escaping mortality risk by leaving the larval habitat outweighs fitness costs of that smaller body size [21]. Alternatively, prey could invest heavily in morphological responses while only slightly reducing activity levels as an anti-predator strategy by delaying metamorphosis and emerging at a larger body size, assuming food acquisition is not lower under predation risk [24]. Therefore, a number of anti-predator strategies are possible under food restriction, including accelerated development with no morphological and/or behavioural response, prey responding either behaviourally or morphologically, or a combination of both behavioural and morphological responses coupled with a longer larval period (slower development rate). Larval period is constrained by the onset of winter for Northern leopard frogs, therefore the trade-off may not necessarily be between starvation and predation risk, but rather metamorphic timing and predation risk – tadpoles that wait too long to transform face mortality from freezing temperatures and/or dessication. Therefore, tadpoles under resource restriction could show a trade-off between behavioural and morphological responses to predation risk, with no cost to development rate, or a trade-off between investment in both defense types and development rate. When there is no energetic constraint, we predict tadpoles will show both morphological and behavioural anti-predator responses, with no corresponding declines in either growth or development rates. Indeed, tadpoles will likely accelerate growth and development in response to higher resource availability, as there are documented benefits from leaving the ponds earlier and/or at a larger size [25], [26].

Using a dragonfly larva-frog tadpole system, we tested the hypothesis that resource limitation constrains prey ability to respond to predation risk. Our null hypothesis is the simple allocation model, which predicts that the expression of phenotypic plasticity will depend on resource availability relative to the level of predation risk. Severely food-restricted tadpoles will show no response to risk, moderately food-restricted tadpoles will express either a morphological or behavioural response to high risk, and food-saturated tadpoles will exhibit responses to predation risk in all traits. Northern leopard frog (*Lithobates pipiens*) tadpoles typically respond to predation risk by decreasing activity [27], [28], [29], [30] and increasing relative tail fin and/or tail muscle depth [28], [29]. While plasticity in life history traits in response to predation risk varies both within and between species [27], [31], [32], we predicted that with no food limitation tadpoles would increase both body size [29] and development speed when under predation risk. In many predator-prey systems, larger body size can reduce vulnerability to predation and larger-bodied prey may acquire more resources by foraging in increasingly risky situations [33], [34]. Attaining larger body size or metamorphosing earlier requires channelling energy into growth and development, which may not be possible for energetically-constrained prey. Statistically, variability in the effect of food and predation risk should be evidenced by a significant interaction term between resource and predation treatments. Energy-mediated trade-offs in prey responses would be evidenced by a negative correlation between the magnitude of behavioural versus morphological responses to predation risk.

## Methods

### Experimental design

We used a 3×3 factorial design to test the effect of both resource level and predation risk intensity on tadpole morphology, life history, and behaviour (n = 6 replicates). Resource level treatments were severe food restriction, moderate restriction, or saturation, corresponding to 2%, 5%, or 20% of the total mass of the tadpoles per aquarium, provided as daily supplementation of crushed algae discs [35]. We selected the above food levels because food rations comprising 2% body mass are known to be severely restricting in tadpoles [36], whereas 20% body mass usually constitutes excessive food [37], [38]. The 5% level was chosen as the intermediate because we sought to detect any non-linearity in tadpole responses to food addition and predation risk, and preliminary work revealed that tadpole responses were highly sensitive to subtle changes in the range of 2–10% body mass (D. Pereira, unpubl.). Predators were dragonfly larvae (*Aeshna* spp.) collected from local ponds. Predators were housed in a separate 40 L bin and fed two leopard frog tadpoles every other day. Tadpoles from many frog species (including *L. pipiens*) can detect chemical cues from predators [39], [40] and exhibit plasticity in morphology and behaviour in response to those cues [41], [42], [43]. Water from the predator bin was added to tadpole aquaria at the same time daily (AM) to create three perceived predation risk level treatments: none = 0 mL predator water +300 mL aged tap water, low = 100 mL predator water +200 mL aged tap water, and high = 300 mL predator water +0 mL aged tap water. Chemical cues of predation persist in the aquatic environment over multiple days [44], therefore tadpoles had a consistent exposure to predation risk over the experimental period.

Northern leopard frog egg masses were collected in April 2009 from ponds near Peterborough, Ontario (44°22′N, 78°03′W). Two broods were reared in 110 L containers to Gosner developmental stage 25 (active, free swimming tadpoles) [45]. Tadpoles were then divided among 54 (9 treatments with 6 replicates each) 12 L glass aquaria filled with approximately 10 L of aged tap water. The laboratory was kept at 18–22°C, and aquaria were cleaned twice weekly by replacing 50% of the water. Each aquarium housed 30 tadpoles, 15 from each brood. This design was strategic in that it allowed us to homogenize the composition of each experimental unit while also introducing genetic variability in the study population. Recent studies have found low heritability in the behavioural response of tadpoles to predation risk [46], [47], and between-population genetic variation appears to largely overshadow within-population variation in phenotypic plasticity, with relatively few sibships from a single pond representing the general population responses [36], [48]. Therefore, our design ensured that results would be both sensitive to experimental manipulation and also generalizable to the broader population.

### Data collection

**Figure 1:**
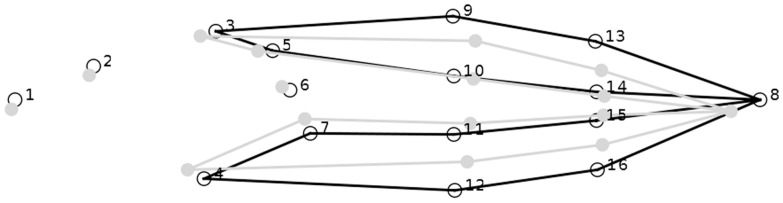
Wireframe drawing of shape change (grey line) against average tadpole shape (black line) described by an increase in the first principal component (PC1) of a Principal Components Analysis (PCA) for all weeks combined using the aquarium averages of sixteen landmarks.

Behavioural data were collected at the same time daily (PM), six days a week, by counting the number of active (tail movement of any kind) and non-active tadpoles per aquarium during 30 second scans. Ten tadpoles were removed each week for morphometric data collection. All ten tadpoles were staged [45] using a dissecting microscope and weighed using a digital balance. Five tadpoles were then photographed at a fixed distance using a Nikon D70 digital camera equipped with a Tamron 90 mm macro 1∶1 lens. Tadpole shape and size was characterized using sixteen landmarks that reflect basic morphological features (figure 1) [43], digitized directly onto each picture, using the software ImageJ (US NIH, Bethesda, MD). We averaged landmark coordinates by aquarium, and then calculated composite shape variables and centroid size (shape-independent size) using a full Procrustes fit in MorphoJ 1.04b software [49]. Tadpoles were returned to their aquarium of origin after data collection.

### Ethics statement

This study was approved by the Trent University Animal Care Committee (Protocol 09013) and strictly followed the Canadian Council on Animal Care's guidelines for ethical animal use. Collection of egg masses and dragonfly larvae was approved through the Ontario Ministry of Natural Resource's Wildlife Scientific Collectors Authorization, and was carried out with landowner permission. Collection did not involve endangered or specially protected species.

### Data analysis

Tadpole mass was log-transformed, and then log(mass), stage, and centroid size were used as dependent variables in three general linear mixed-effects models, with predation risk, resource level, and week as fixed factors, and aquarium as a random factor. Developmental stage was treated as a continuous variable [50], [51]. To look at tadpole shape, we conducted a Principal Components Analysis (PCA) for all weeks combined using the aquarium averages of the shape variables in MorphoJ 1.04b software [49]. Based on examination of the scree diagram [52], we took the first three principal components, explaining cumulatively over 80% of the variation in the data, and used them (PC1, PC2, PC3) as dependent variables in general linear mixed effects models with predation risk, resource level, and week as fixed factors, and aquarium as a random factor. Data met the assumptions of normality and homeoscedacity for these tests. We used Bonferroni correction for analyses of mass and centroid size, as they both reflect tadpole size (corrected α = 0.025) and for analyses of tadpole shape (PC1, PC2, and PC3) since they are all measurements of shape change (corrected α = 0.017). Significance was set at α = 0.05 for analyses of tadpole activity and developmental stage. Behavioural data were proportional and therefore activity was used as a dependent variable (with binomial error distribution) in a generalized linear mixed effects model, with predation risk, resource level, and week as fixed factors, and aquarium as a random factor, using the R statistical package ‘lme4’ [53]. Significance in the generalized linear mixed effects model was determined using a type III test of fixed effects (α = 0.05) analysis of variance in the R statistical package ‘MixMod’ [54].

We conducted a series of three correlation analyses to assess whether the relationship between behavioural (activity) and morphological (centroid size, shape) responses to predation risk changed at different resource levels. Behavioural data were arcsine square root transformed to meet the assumption of normality. PC1 was used as the measurement of tadpole shape, as it was the only principal component that varied significantly with predation risk (table 1). We used response data from all three weeks and risk levels so that we could detect trade-offs across the whole spectrum of both time and predation risk that was encompassed by our experimental protocols. Relationships were assessed using Pearson's product-moment correlation (*r*) and significance was set at α = 0.025 after Bonferroni correction for multiple comparisons. Differences between slopes of the relationships were assessed using a t-test [55]. Mixed effects models were run using program R (R Development Core Team 2011). Correlations and graphs were made using Statistica 7.0 (StatSoft 2004).

**Table 1:** Univariate results of a factorial ANOVA using the first three principal components from PCAs on Northern leopard frog (*Lithobates pipiens*) tadpole shape variables, over three weeks of experimentation at three resource level treatments (2%, 5%, and 20% of total tadpole mass per aquarium given as food every other day) and three predation risk level treatments (0 mL, 100 mL, and 300 mL predator-treated water added daily).

	*F*	d.f.	*P*
*PC1*			
**Resource Level**	**10.1**	**2, 45**	**0.000**
**Predation Risk**	**19.5**	**2, 45**	**0.000**
Resource x Predation	1.4	4, 45	0.248
**Week**	**647.4**	**2, 90**	**0.000**
**Predation x Week**	**32.0**	**4, 90**	**0.000**
**Resource x Week**	**7.2**	**4, 90**	**0.000**
Predation x Resource x Week	1.9	8, 90	0.076
*PC2*			
Resource Level	2.5	2, 45	0.094
Predation Risk	2.5	2, 45	0.092
Resource x Predation	0.2	4, 45	0.912
Week	0.1	2, 90	0.941
Predation x Week	1.3	4, 90	0.267
**Resource x Week**	**22.8**	**4, 90**	**0.000**
Predation x Resource x Week	1.1	8, 90	0.345
*PC3*			
Resource Level	1.6	2, 45	0.220
Predation Risk	4.2	2, 45	0.021
Resource x Predation	0.7	4, 45	0.730
**Week**	**11.0**	**2, 90**	**0.000**
**Predation x Week**	**3.9**	**4, 90**	**0.006**
**Resource x Week**	**5.4**	**4, 90**	**0.001**
Predation x Resource x Week	0.8	8, 90	0.625

Significant results are in bold.

## Results

### Growth and development

**Figure 2:**
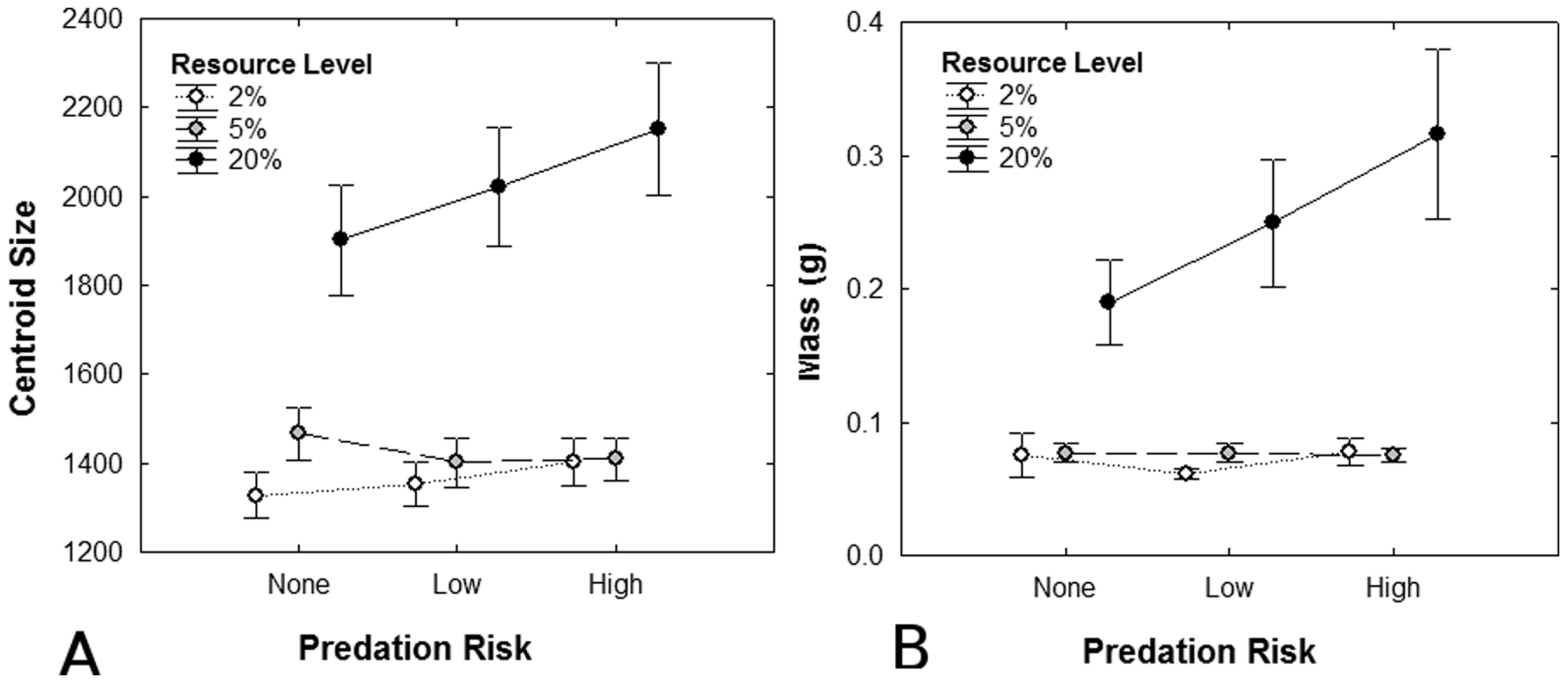
Interaction plots of resource level (2%: severe restriction, 5%: moderate restriction, 20%: saturation) and predation risk (none, low, high) on mean (±95% CI) A: Centroid Size and B: Mass (g) of Northern leopard frog (*Lithobates pipiens*) tadpoles.

**Figure 3:**
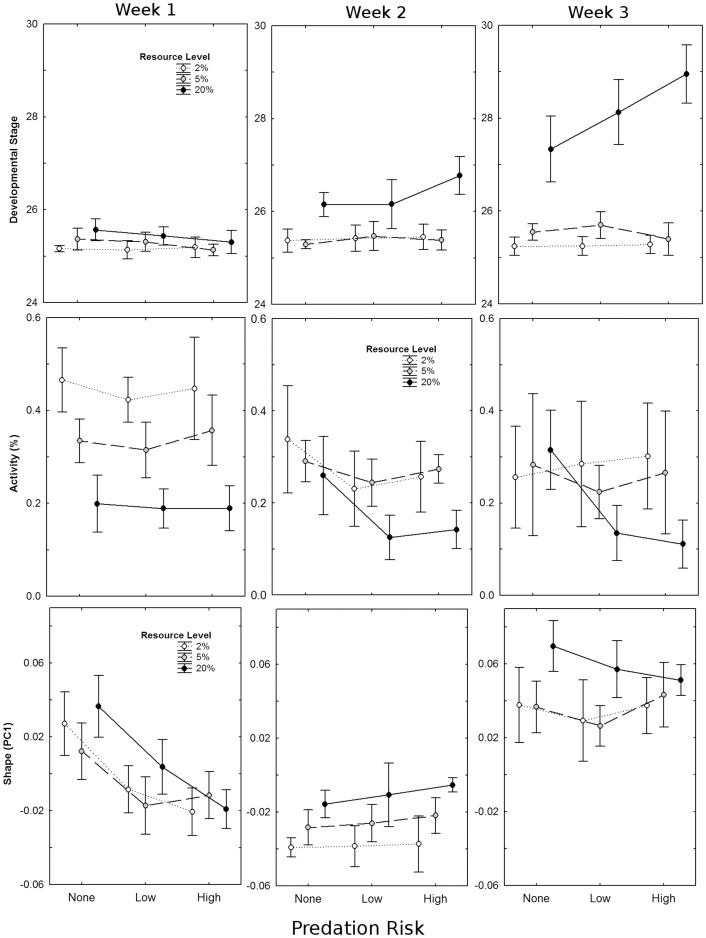
Interaction plots of resource level (2%: severe restriction, 5%: moderate restriction, 20%: saturation) and predation risk (none, low, high) on mean (±95% CI) developmental stage (top), activity (proportion of active tadpoles per aquarium; middle), and shape (PC1; bottom) of Northern leopard frog (*Lithobates pipiens*) tadpoles averaged by week, across three weeks of experimentation.

Tadpoles at the saturation level of resource availability were significantly heavier, larger, and further developed than those at either moderate or severe food restriction (*P*<0.0001, in all cases). There was an interaction between predation risk and resource level on tadpole centroid size (*F*_4, 45_ = 5.7, *P* = 0.0008) and mass (*F*_4,45_ = 3.2, *P* = 0.021); only tadpoles at saturation responded to predation risk with increasing levels of predation risk resulting in increases in body size (mass: *F*_2,45_ = 5.2, *P* = 0.009; centroid size: *F*_2,45_ = 5.9, *P* = 0.005; figure 2). There was also an interaction between resource availability and week on all measured traits (*P*<0.0001, in all cases), with tadpoles showing the greatest increase in size and development during the third week of the experiment. Development was accelerated in response to predation risk (*F*_2,45_ = 5.0, *P* = 0.011), and was influenced by a 3-way interaction between predation risk, resource level, and experimental week (*F*_8, 90_ = 5.1, *P*<0.0001). Tadpoles showed no response to predators during week 1, response to the highest predation risk level only from tadpoles at resource saturation during week 2, and an increasing response to both low and high predation risk, but again only from tadpoles at resource saturation, during week 3 (figure 3, top). Neither mass (*F*_8,90_ = 1.0, *P* = 0.44) nor centroid size (*F*_8,90_ = 1.4, *P* = 0.21) showed a similar three-way interaction.

### Behaviour

In general, tadpoles decreased activity with increasing predation risk (*F*_1,1016_ = 602.2, *P* = 0.01) but not resource availability (*F*_1,1016_ = 0.01, *P* = 0.92); however, these effects varied depending on the experimental week (Resource x Week: *F*_1,1016_ = 4.83, *P* = 0.03; Predation x Week: *F*_1,1016_ = 27.70, *P*<0.001). Resource availability influenced activity level during the first week of the experiment, whereas predation risk effects were more pronounced during the second and third weeks (figure 3, middle). There was no significant interaction between predation risk and resource availability (*F*_1,1016_ = 0.10, *P* = 0.75), nor was there a three-way interaction between predation risk, resource availability, and week (*F*_1,1016_ = 0.02, *P* = 0.88).

### Morphology

PC1 (variance explained = 46.2%) described tadpoles with shallower tails at higher resource availability, and deeper tails with increased predation risk (figure 3, bottom). There were significant interactions between week and predation risk as well as between week and resource availability on PC1 (table 1). Tadpoles during the first week of the experiment responded to predation risk by decreasing PC1 (developing shorter, deeper tails), but showed no clear differences between resource treatments. After the second week, the effect of predation risk was lost, but increasing resource availability caused an increase along PC1, with tadpoles having relatively longer, shallower tails. Finally, by the end of week three, effects of both predation risk and resource availability were mostly lost, though tadpoles in the resource saturation treatment still tended to have relatively longer, shallower tails (figure 3, bottom). PC2 and PC3 did not vary significantly with either predation risk or resource availability (table 1).

### Trade-offs

**Figure 4:**
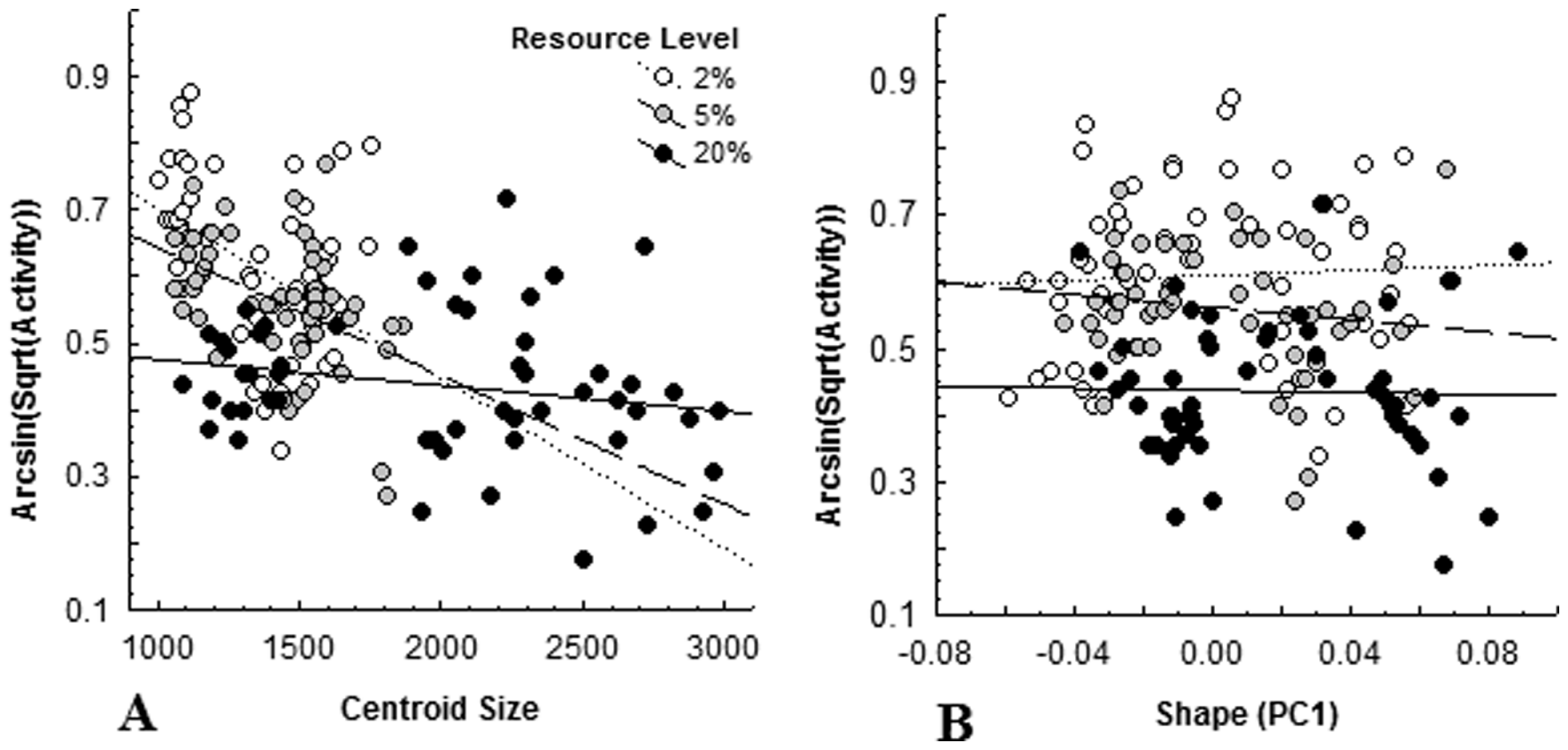
Relationship between behavioural (activity (%)) and morphological (A: centroid size, B: shape (PC1)) responses of Northern leopard frog tadpoles (*Lithobates pipiens*). to variation in predation risk averaged by week, across three weeks of experimentation, at three resource level treatments (2%: severe restriction, 5%: moderate restriction, 20%: saturation).

There was no evidence of a relationship between activity and tadpole shape at any resource level (2%: *r* = −0.02; 5%: *r* = −0.09; 20%: *r* = −0.21; all *P*>0.13; figure 4A). Tadpoles under both severe (*r* = −0.41, *P* = 0.002) and moderate (*r* = −0.45, *P*<0.001) food restriction showed a significant negative relationship between activity level and body size, but this relationship was not found at food saturation (*r* = −0.20, *P* = 0.14; figure 4B). The slope of the saturation relationship differed from both moderate (*t*_104_ = 2.39, *P* = 0.019) and severe (*t*_104_ = −2.73, *P* = 0.008) food restriction, which, in turn, were not different from each other (*t*_104_ = −0.67, *P* = 0.50).

## Discussion

Our data supported the simple allocation model - tadpoles at saturation increased body size, decreased activity, and accelerated development in response to predation risk whereas tadpoles under food restriction failed to show similar responses to increased risk. Body size and activity were negatively correlated under restricted resources but not at saturation, however, we found no evidence of a trade-off between tadpole shape and activity at any resource level. We also noted a significant effect of time on direction and magnitude of behavioural, morphological, and developmental responses. Contrary to previous studies, which generally show either an increasing or steady morphological response over exposure time [57], [58], [59], tadpoles altered tail shape in response to predation risk only during the first week of the experiment. During the second and third weeks of the experiment, only prey at resource saturation responded to predation risk by increasing growth and development rates while lowering activity levels.

The lack of difference in response to predation risk between our moderate and severe restriction treatments (2% and 5% of body mass fed daily as crushed algae discs, respectively) is puzzling. Tadpoles from a number of species, including Northern leopard frogs, have been shown to respond to predation risk both morphologically and behaviourally at resource levels comparable to our treatments (2% and 4% body mass/day) [60], [61]. In both those studies, predators were housed in cages held within tadpole enclosures [60], [61]; it is possible that by adding cue water, perceived predation risk was too low (due to dilution or a lack of visual cues) to elicit a response at restricted resource level. Therefore, trade-offs between defense types, and investment in defense versus life history under food restriction, are likely to be manifested only under high levels of predation risk. However, in our experiment, tadpoles at saturation did respond to the addition of cue water, and the magnitude of the response increased between the low and high predation risk treatments, demonstrating that tadpoles were capable of detecting and responding to differences in the cue concentrations. Furthermore, our resource saturation treatment (20%) is comparable to other studies of resource availablity on tadpole response (16%–18%) [60], [61], and our growth and development rates at resource saturation are similar to published data for Northern leopard frog under ad lib conditions (15%) [62] as well as for wild populations [63], [64]. Therefore, we are confident that responses found in our experiment at resource saturation represent biologically significant defenses of prey to predation risk.

Despite being a relatively short experiment (three weeks), we found significant temporal variation in activity, tail morphology, and development in response to both predation risk and resource availability. Tadpoles in the first week had relatively deeper tails but showed no change in behaviour in response to predation risk; however, they did decrease activity levels in response to increasing resource availability. From the literature we would expect a higher magnitude of behavioural response to predation risk earlier in development at smaller, more vulnerable, body sizes [57], [58], [59]. Stage-specific responses to predation risk are thought to reflect threat-sensitivity by prey in situations where predation risk varies with body size or developmental stage [65], [66]. For anuran larvae, plastic responses may also be constrained by developmental capabilities during ontogeny [67]. Alternatively, morphological defenses are predicted to be insenstive to resource availability in models that allow for flexibility in developmental timing and size at metamorphosis [21]. That is, morphological responses will be favoured if those defenses allow prey to stay in the larval environment longer and attain a larger size at metamorphosis. Such a response has been noted in *Rana temporaria*, where tadpoles increased relative tail depth in response to predator presence regardless of competitor density [68]. Our data support this model, but only in the first week of the experiment, after which the morphological response to predators is lost and a behavioural response (decreasing activity level) is gained under resource saturation.

In theory, decreasing activity in response to predation risk results in declining rates of growth and/or development [67], [69], [70]. However, this conflict between growth and predation risk is not a universal phenomenon, with some studies revealing no decrease in growth or development despite lower activity levels in response to predation risk [28], [71]. Changes in body size in response to predation risk may be an artefact of experimental venue [72] and body size is considered in some studies to be a non-adaptive response to predation risk [7]. Conversely, the acceleration of growth could be adaptive because size matters to survival when predators are gape-limited [73], [74]. In amphibian systems, smaller tadpoles face higher predation risk from dragonfly larvae than do larger tadpoles [75], [76], and relatively larger dragonfly larvae are more effective at capturing and consuming prey [77]. Northern leopard frogs can increase body size in response to predation risk despite a concurrent decrease in all activities, including foraging [27], [30], suggesting body size in this species may be a defensive trait. While we cannot determine the adaptive value of body size based on our experimental results, we did find that tadpoles at resource saturation increased mass in response to predation risk, showing that conflict between growth allocation and predation risk avoidance becomes inconsequential if resources are sufficiently abundant.

The importance of body size is considered further in our trait correlation analysis. Under resource restriction, but not saturation, we found a negative relationship between tadpole activity and body size. We cannot determine the causal direction of this relationship from our data, that is, whether larger tadpoles become less active or less active tadpoles also grow larger. We also know of no previous study that has documented a comparable change in the relationship between behavioural and morphological anti-predator responses under different levels of resource availability. Foraging theory suggests that once an animal's basic nutritional needs are met, its risk sensitivity increases which, in turn, leads to lower activity [1], [6]. It is possible that tadpoles must reach a threshold body size before altering activity rates in response to predation risk, and that the negative correlation we found represents the relationship between body size and a behavioural response to risk. We are unable, however, to discount the physiological explanation that tadpoles that are less active expend less energy and are therefore able to grow larger. Yet, this explanation is not entirely satisfying as we expect the relationship to also be present at resource saturation, which it was not, and activity in our study was defined as all movement, including feeding behaviours. Furthermore, we found a significant effect of time on behavioural response: tadpoles decreased activity levels in response to predation risk only during the second and third weeks of the experiment. This too provides support for the hypothesis that tadpoles must reach a minimum body size before responding behaviourally to perceived risk.

The simple allocation model was supported by our data, however, we were unable to distinguish defensive responses between severe and moderate resource restriction treatments and we found significant temporal variation in both the type and magnitude of defensive response. The importance of body size as either a confouding variable or anti-predator strategy is highlighted by our finding that, during the first week of the experiment when tadpoles were at their smallest body sizes, prey showed only a morphological, not behavioural, response to predation risk. However, during the second and third weeks of the experiment, tadpoles increased body size and development rate while decreasing activity level in response to predators, but lost any differences in shape. Body size was also negatively related to activity level, but only when resources were restricted, suggesting that body size may play a role in determining the deployment of behavioural defenses. Accordingly, there is a need to more fully quantify the adaptive value of body size as a function of both predator defense and as a life history trait. Further studies are needed to determine how higher levels of predation risk interact with resource availability over the entire length of the larval period. Our results provide an important starting point for understanding how interactions between condition and extrinsic risk factors may play a critical role in the production of inducible defenses over time.

## Acknowledgments

We are grateful to Kristen Landolt and Brian Atkins for lab, field, and husbandry assistance, Nic Robar for help with egg collection, Richard Feldman for his helpful advice regarding statistical analysis, Matthew Keevil, the Murray lab, and anonymous reviewers for constructive criticism of the manuscript.

## References

[1] McNamara JM, Houston AI (1987) Starvation and predation as factors limiting population size. Ecology 68: 1515–1519.

[2] Lima SL, Dill LM (1990) Behavioral decisions made under the risk of predation: a review and prospectus. Canadian Journal of Zoology 68: 619–640 10.1139/z90-092)

[3] Sinclair ARE, Arcese P (1995) Population consequences of predation-sensitive foraging: the serengeti wildebeest. Ecology 76: 882–891.

[4] Houston A, Clark C, McNamara J, Mangel M (1988) Dynamic models in behavioural and evolutionary ecology. Nature 332: 29–34.

[5] Harvell CD (1990) The ecology and evolution of inducible defenses. The Quarterly Review of Biology 65: 323–340. 2236483 10.1086/416841

[6] Werner EE, Anholt BR (1993) Ecological consequences of the trade-off between growth and mortality rates mediated by foraging activity. The American Naturalist 142: 242–272. 10.1086/285537 19425978

[7] Steiner UK, Pfeiffer T (2007) Optimizing time and resource allocation trade-offs for investment into morphological and behavioral defense. The American Naturalist 169: 118–129. 10.1086/509939 17206590

[8] Barry MJ (1994) The costs of crest induction for *Daphnia carinata* . Oecologia 97: 278–288. 28313940 10.1007/BF00323161

[9] Schmitz OJ (1998) Direct and indirect effects of predation and predation risk in old-field interaction webs. The American Naturalist 151: 327–342 10.1086/286122) 18811324

[10] Downes S (2001) Trading heat and food for safety: costs of predator avoidance in a lizard. *Ecology* 82: , 2870–2881.

[11] Pettersson LB, Brönmark C (1999) Energetic consequences of an inducible morphological defense in crucian carp. Oecologia 121: 12–18 10.1007/s004420050901) 28307880

[12] Van Buskirk J (2000) The costs of an inducible defense in anuran larvae. Ecology 81: 2813–2821.

[13] Hammill E, Rogers A, Beckerman AP (2008) Costs, benefits and the evolution of inducible defenses: a case study with *Daphnia pulex* . Journal of Evolutionary Biology 21: 705–715. 18355186 10.1111/j.1420-9101.2008.01520.x

[14] Lima SL, Valone TJ, Caraco T (1985) Foraging-efficiency-predation-risk trade-off in the grey squirrel. Animal Behaviour 33: 155–165 10.1016/S0003-3475(85)80129-9)

[15] Kohler SL, McPeek MA (1989) Predation riska and the foraging behavior of competing stream insects. Ecology 70: 1811–1825.

[16] Cowlishaw G (1997) Trade-offs between foraging and predation risk determine habitat use in a desert baboon population. Animal Behaviour 53: 667–686 10.1006/anbe.199.0298) 9268454

[17] Stoks S (1999) Autotomy shapes the trade-off between seeking cover and foraging in larval damselflies. Behavioural Ecology and Sociobiology 47: 70–75 10.1007/s002650050651)

[18] Cooper WE (2003) Shifted balance of risk and cost after autotomy affects use of cover, escape, activity, and foraging in the keeled earless lizard (*Holbrookia propinqua*). Behavioural Ecology and Sociobiology 54: 179–187 10.1007/s00265-003-0619-y)

[19] Cooper WE Jr (2007) Compensatory changes in escape and refuge use following autotomy in the lizard *Sceloperus virgatus* . Canadian Journal of Zoology 85: 99–107 10.1139/z06-200)

[20] Anholt BR, Werner EE (1995) Interaction between food availability and predation mortality mediated by adaptive behavior. Ecology 76: 2230–2234.

[21] Higginson AD, Ruxton GD (2009) Dynamic models allowing for flexibility in complex life histories accurately predict timing of metamorphosis and antripredator strategies of prey. Functional Ecology 23: 1103–1113.

[22] Brookes JI, Rochette R (2007) Mechanism of a plastic phenotypic response: predator-induced shell thickening in the intertidal gastropod *Littorina obtusata* . Journal of Evolutionary Biology 20: 1015–1027 10.111/j.1420-9101.2007.01299.x) 17465912

[23] Relyea RA, Auld JR (2004) Having the guts to compete: how intestinal plasticity explains costs of inducible defences. Ecology Letters 7: 869–875 10.1111/j.1461-0248.2004.00645.x)

[24] Higginson AD, Ruxton GD (2010) Adaptive changes in size and age at metamorphosis can qualitatively vary with predator type and available defenses. Ecology 91: 2756–2768. 20957968 10.1890/08-2269.1

[25] Beck CW, Congdon JD (2000) Effects of age and size at metamorphosis on performance and metabolic rates of Southern Toad, *Bufo terrestris*, metamorphs. Functional Ecology 14: 32–38.

[26] Altwegg R, Reyer H-U (2003) Patterns of natural selection on size at metamorphosis in water frogs. Evolution 57: 872–882. 12778556 10.1111/j.0014-3820.2003.tb00298.x

[27] Relyea RA (2000) Trait-mediated indirect effects in larval anurans: Reversing competition with the threat of predation. Ecology 81: 2278–2289 10.1890/0012-9658(2000)0812278:TMIEIL2.0.CO;2)

[28] Relyea RA (2001) Morphological and behavioral plasticity of larval anurans in response to different predators. Ecology 82: 523–540 10.1890/0012-9658(2001)0820523:MABPOL2.0.CO;2)

[29] Hossie TJ, Ferland-Raymond B, Burness G, Murray DL (2010) Morphological and behavioural responses of frog tadpoles to perceived predation risk: A possible role for corticosterone mediation? Écoscience 17: 100–108 10.2980/17-1-3312)

[30] Hossie TJ, Murray DL (2010) You can’t run but you can hide: refuge use in frog tadpoles elicits density-dependent predation by dragonfly larvae. Oecologia 163: 395–404. 20130916 10.1007/s00442-010-1568-6

[31] Laurila A, Kujasalo J, Ranta J (1998) Predator induced changes in life history in two anurans: effects of predator diet. Oikos 83: 307–317.

[32] Koprivnikar J (2010) Interactions of environmental stressors impact survival and development of parasitized larval amphibians. Ecological Applications 20: 2263–2272. 21265456 10.1890/09-1558.1

[33] Johnsson JI (1993) Big and brave: Size selection affects foraging under risk of predation in juvenile rainbow trout, *Oncorhynchus mykiss* . Animal Behaviour 45: 1219–1225 10.1006/anbe.1993.1143)

[34] Rochette R, Himmelman JH (1996) Does vulnerability influence trade-offs made by whelks between predation risk and feeding opportunities? Animal Behaviour 52: 783–794 10.1006/anbe.1996.0223)

[35] Venesky MD, Wassersug RJ, Parris MJ (2010) The impact of variation in labial tooth number on the feeding kinematics of tadpoles of Southern leopard frog (*Lithobates sphenocephalus*). Copeia 2010: 481–486.

[36] Werner EE (1992) Competitive interactions between wood frog and northern leopard frog larvae: The influence of size and activity. Copeia 1992: 157–169.

[37] Anholt BR, Werner EE, Skelly DK (2000) Effect of food and predators on the activity of four larval ranid frogs. Ecology 81: 3509–3521 10.1890/0012-9658(2000)0813509:EOFAPO2.0.CO;2)

[38] Glennemeier K, Denver RJ (2002) Role for corticoids in mediating the response of *Rana pipiens* tadpoles to intraspecific competition. Journal of Experimental Zoology 292: 32–40 10.1002/jez.1140) 11754020

[39] Stouffer H-P, Semlitsch RD (1993) Effects of visual, chemical, and tactile cues of fish on the behavioural responses of tadpoles. Animal Behaviour 46: 355–364 10.1006/anbe.1993.1197)

[40] Eklöv P (2000) Chemical cues from multiple predator-prey interactions induce changes in behavior and growth of anuran larvae. Oecologia 123: 192–199 10.1007/s004420051005) 28308723

[41] Petranka JW, Kats LR, Sih A (1987) Predator-prey interactions among fish and larval amphibians: use of chemical cues to detect predatory fish. Animal Behaviour 35: 420–425 10.1016/S0003-3472(87)80266-X)

[42] Petranka JW, Hayes L (1998) Chemically mediated avoidance of a predatory odonate (*Anax junius*) by American toad (*Bufo americanus*) and wood frog (*Rana sylvatica*) tadpoles. Behavioural Ecology and Sociobiology 42: 263–271 10.1007/s002650050438)

[43] Ferland-Raymond B, Murray DL (2008) Predator diet and prey adaptive responses: Can tadpoles distinguish between predators feeding on congeneric vs. conspecific prey? Can.adian Journal of Zoology 86: 1329–1336 10.1139/Z08-117)

[44] Peacor SD (2006) Behavioural response of bullfrog tadpoles to chemical cues of predation risk are affected by cue age and water source. Hydrobiologia 573: 39–44 10.1007/s10750-006-0256-3)

[45] Gosner KL (1960) A simplified method for staging anuran embryos and larvae with notes on identification. Herpetologica 16: 183–190.

[46] Relyea RA (2005) The heritability of inducible defenses in tadpoles. Journal of Evolutionary Biology 18: 856–866 10.1111/j.1420-9101.2005.00882.x) 16033557

[47] Watkins TB, McPeek MA, Fox S, Wood R (2006) Growth and predation risk in green frog tadpoles (*Rana clamitans*): A quantitative genetic analysis. Copeia 2006: 478–488 10.1643/0045-8511(2006)2006478:GAPRIG2.0.CO;2)

[48] Relyea RA (2002) Local population differences in phenotypic plasticity: predator induced changes in wood frog tadpoles. Ecological Monographs 72: 77–93 10.1890/0012-9615(2002)0720077:LPDIPP2.0.CO;2)

[49] Klingenberg CP (2011) MorphoJ: an integrated software package for geometric morphometrics. Molecular Ecology Resources 11: 353–357 10.1111/j.1755-0998.2010.02924.x) 21429143

[50] Laurila A, Karttunen S, Merilä J (2002) Adaptive phenotypic plasticity and genetics of larval life histories in two *Rana temporaria* populations. Evolution 56: 617–627 10.1111/j.0014-3820.2002.tb01371.x) 11989690

[51] Ireland DH, Wirsing AJ, Murray DL (2007) Phenotypically plastic responses of green frog embryos to conflicting predation risk. Oecologia 152: 162–168 10.1007/s00442-006-0637-3) 17216211

[52] Quinn GP, Keough MJ (2002) Experimental Design and Data Analysis for Biologists. New York: Cambridge University Press.

[53] Bates M, Maechler M, Bolker B (2011) lme4: Linear mixed-effects models using S4 classes. R package version 0.999375-42. Available: http://CRAN.R-project.org/package=lme4. Accessed 2012 October 22.

[54] Kuznetsova A, Brockhoff PB (2012) MixMod: Analysis of Mixed Models. R package version 1.0. Available: http://CRAN.R-Project.org/package=MixMod. Accessed 2013 Jan 7.

[55] Armitage P (1980) Statistical Methods in Medical Research. Oxford: Blackwell Scientific Publications.

[56] Van Buskirk J (2002) Phenotypic lability and the evolution of predator-induced plasticity in tadpoles. Evolution 56: 361–370 10.1111/j.0014-3820.2002.tb01346.x) 11926504

[57] Relyea RA (2003) Predators come and predators go: The reversibility of predator-induced traits. Ecology 84: 1840–1848 10.1890/0012-9658(2003)0841840:PCAPGT2.0.CO;2)

[58] Hossie TJ, Murray DL (2012) Assessing behavioural and morphological responses of frog tadpoles to temporal variability in predation risk. Journal of Zoology 288: 275–282 10.1111/j.14697998.2012.00955.x)

[59] Anholt BR, Skelly DK, Werner EE (1996) Factors modifying antipredator behavior in larval toads. Herpetologica 52: 301–313 10.1007/BF00317907)

[60] Anholt BR, Werner E, Skelly DK (2000) Effect of food and predators on the activity of four larval ranid frogs. Ecology 81: 3509–3521.

[61] Steiner UK (2007) Investment in defense and cost of predator-induced defense along a resource gradient. Oecologia 152: 201–210 10.1007/s00442-006-0645-3) 17221255

[62] Allran JW, Karasov WK (2000) Effects of atrazine and nitrate on Northern leopard frog (*Rana pipiens*) larvae exposed in the laboratory from posthatch through metamorphosis. Environmental Toxicology and Chemistry 19: 2850–2855.

[63] Froom B (1982) Amphibians of Canada. Toronto: McClelland and Stewart Limited. 120 p.

[64] Cook FR (1984) Introduction to Canadian amphibians and reptiles. Ottawa: National Museums of Canada. 200 p.

[65] Puttlitz MH, Chivers DP, Keisecker JM, Blaustein AR (1999) Threat-sensitive predator avoidance by larval pacific treefrogs (Amphibia, Hylidae). Ethology 105: 449–456 10.1046/j.1439-0310.1999.00416.x)

[66] Danner BJ, Joern A (2003) Stage-specific behavioral responses of *Ageneotettix deorum* (Orthoptera: Acrididae) in the presence of lycosid spider predators. Journal of Insect Behaviour 16: 453–464 10.1023/A:1027337405495)

[67] Laurlia A, Kujasalo J (1999) Habitat duration, predation risk and phenotypic plasticity in common frog (*Rana temporaria*) tadpoles. Journal of Animal Ecology 68: 1123–1132 10.1046/j.1365-2656.1999.00354.x)

[68] Teplitsky C, Laurila A (2007) Flexible defense strategies: Competition modifies investment in behavioral vs. morphological defenses. Ecology 88: 1641–1646 10.1890/06-1703.1) 17645010

[69] Skelly DK (1992) Field evidence for a cost of behavioral antipredator response in a larval amphibian. Ecology 73: 704–708.

[70] Schoeppner NM, Relyea RA (2005) Damage, digestion, and defence: the roles of alarm cues and kairomones for inducing prey defences. Ecology Letters 8: 505–512 10.1111/j.1461-0248.2005.00744.x) 21352454

[71] Van Buskirk J, Arioli M (2002) Dosage response of an induced defense: How sensitive are tadpoles to predation risk? Ecology 83: 1580–1585 10.1890/0012-9658(2002)0831580:DROAID2.0.CO;2)

[72] Winkler JD, Van Buskirk J (2012) Influence of experimental venue on phenotype: multiple traits reveal mulitple answers. Functional Ecology 26: 513–521.

[73] Nilsson PA, Bronmark C (2000) Prey vulnerability to a gape-size limited predator: Behavioural and morphological impacts on Northern pike piscivory. Oikos 88: 539–546.

[74] Mathis A, Murray KL, Hickman CR (2003) Do experience and body size play a role in responses of larval ringed salamanders, *Ambystoma annulatum*, to predator kairomones? Laboratory and field assays. Ethology 109: 159–170.

[75] Travis J, Keen WH, Juilianna J (1985) The role of relative body size in a predator-prey relationship between dragonfly naiads and larval anurans. Oikos 45: 59–65.

[76] Semlitsch RD (1990) Effects of body size, sibship, and tail injury on the susceptibility of tadpoles to dragonfly predation. Canadian Journal of Zoology 68: 1027–1030.

[77] Babbitt KJ, Tanner GW (1998) Effects of cover and predator size on survival and development of *Rana utricularia* tadpoles. Oecologia 114: 258–262 10.1007/s004420050444) 28307940

